# Anti-tumor enhancement of Fei-Liu-Ping ointment in combination with celecoxib via cyclooxygenase-2-mediated lung metastatic inflammatory microenvironment in Lewis lung carcinoma xenograft mouse model

**DOI:** 10.1186/s12967-015-0728-1

**Published:** 2015-11-23

**Authors:** Rui Liu, Honggang Zheng, Weidong Li, Qiujun Guo, Shulin He, Yoshiro Hirasaki, Wei Hou, Baojin Hua, Conghuang Li, Yanju Bao, Yebo Gao, Xin Qi, Yingxia Pei, Yun Zhang

**Affiliations:** Department of Oncology, Guang’anmen Hospital, China Academy of Chinese Medical Sciences, Number 5 Beixiange, Xicheng District, Beijing, 100053 China; Cancer Institute, China Academy of Chinese Medical Sciences, Beijing, 100053 China; Beijing University of Chinese Medicine, Beijing, 100029 China; Department of Japanese-Oriental (Kampo) Medicine, Graduate School of Medicine, Chiba University, 1-8-1 Inohana, Chuo-ku, Chiba, 260-8670 Japan

**Keywords:** Lung cancer, Fei-Liu-Ping ointment, Celecoxib, Inflammatory microenvironment

## Abstract

**Background:**

Fei-Liu-Ping (FLP) ointment is an oral prescription medication that has been widely applied to treat lung cancer patients in China. Regulation of the metastatic microenvironment is an important therapeutic approach for prevention and treatment of tumor recurrence and metastasis. The advantage of Traditional Chinese Medicine management of lung cancer lies in the prevention of recurrence and metastasis. Our previous study has demonstrated that FLP ointment could regulate lung inflammatory microenvironment in vitro. However, the effects of FLP on the tumor microenvironment in vivo are still poorly understood. The objective of this study is to investigate the effect of FLP alone or in combination with celecoxib in the prevention of lung cancer progression by Cyclooxygenase (Cox)-2 mediated tumor inflammatory microenvironment in vivo.

**Methods:**

120 Lewis lung carcinoma xenograft mice were divided equally into four groups: vehicle, FLP, celecoxib, and FLP plus celecoxib. The dynamic growth of the xenografted tumors was observed using an in vivo fluorescence imaging system. Mice were sacrificed on day 14, day 21, and day 28, and tumor specimens and lung tissues were harvested to detect the metastasis-associated protein expression.

**Results:**

Tumor inhibition rate was 15.4, 44.2, 47.4 % at day 14, 37.3, 34.7, 61.5 % at day 21, and 15.5, 10.3, 32.5 % at day 28 after treatment of FLP, celecoxib, and FLP plus celecoxib, respectively. Upon treatment of FLP and celecoxib together, lung metastasis rate was 30 % (8 metastatic nodules) lower than other groups. FLP inhibited Cox-2 expression in a time-dependent manner. Moreover, FLP inhibited N-cadherin, matrix metalloproteinases (MMP)-9, and Vimentin expression. Treatment of FLP in combination with celecoxib was more effective than FLP or celecoxib alone in inhibiting vascular endothelial growth factor, platelet-derived growth factor receptors β, microsomal Prostaglandin E synthase-1, MMP-2, MMP-9, N-cadherin, and Vimentin expression, but increased E-cadherin expression.

**Conclusions:**

FLP inhibited tumor growth and metastasis in a Lewis lung xenograft mice model through the Cox-2 pathway. FLP in combination with celecoxib enhanced the antitumor growth and anti-metastasis effects. Traditional Chinese herbs combined with anti-inflammatory drugs might offer a promising strategy to prevent tumor metastasis.

## Background

Lung cancer is the leading cause of cancer-related deaths in the world [1]. Despite advances in surgical techniques, chemotherapy, and radiotherapy, the 5-year survival rate for lung cancer patients is lower than 20 % [2]. The high mortality is likely attributed to early metastasis. Metastatic spread is the critical cause of mortality and treatment failure [3, 4]. Therefore, development of new therapies to treat patients with metastatic lung cancer is necessary to reduce mortality rates. Prevention of recurrence and metastasis is an important step towards management for lung cancer patients. Research on lung cancer has recently focused on tumor microenvironment and recognized that alterations in the tumor microenvironment are a predictor of tumor occurrence and metastasis [5]. Various agents targeting the tumor inflammatory microenvironment provide a new therapeutic method for preventing tumor metastasis [6]. Changes in the tumor microenvironment occur slowly, and has certain correlation with maintenance therapy to prevent tumor recurrence and metastasis. Currently, chemotherapeutic drugs and targeted agents are commonly used in maintenance therapy. Pemetrexed and Erlotinib are widely used as maintenance therapy for advanced non-small cell lung cancer [7, 8]; however, their adverse effects and high economic burden reduce patient compliance [9].

Tumor microenvironment plays a critical role in tumor growth, progression, and metastasis [10]. It is increasingly recognized that tumor inflammatory microenvironment is closely related to tumor metastasis [11–15]. Cyclooxygenase-2 (Cox-2) is a rate-limiting enzyme for prostanoid biosynthesis, and plays a crucial role in the tumor inflammatory microenvironment [16, 17]. Cox-2 is over-expressed in all the metastatic process of cancer and involved in angiogenesis, epithelial-mesenchymal transition (EMT) initiation, and extracellular matrix (ECM) destruction [18–21]. Increasing evidence has shown that the Cox-2 gene was constitutively over-expressed in most human solid tumors, such as colon cancer, breast cancer, and lung cancer, and could serve as a predictor of poor prognosis [22–24].

Inflammation is necessary to promote cancer initiation and progression through angiogenesis and change of tumor microenvironment [25]. Anti-inflammatory drugs have become indispensable for cancer prevention and treatment. Indeed, long-term use of non steroidal anti-inflammatory drugs (NSAIDs), such as aspirin, were shown to decrease the risk of colon cancer by 40–50 % [26]. Cox-2 inhibitors are amongst the most popular medications worldwide. The US Food and Drug Administration (FDA) approved celecoxib, which is a hallmark of COX-2 inhibitors used for tumor prevention, as the adjuvant therapy for treatment of familial adenomatous polyposis in 1999 [27].

Traditional Chinese Medicine (TCM), as an important part of complementary and alternative therapy, exhibit advantages in the prevention of tumor recurrence and metastasis compared with radiotherapy and chemotherapy [28]. TCM is often used for maintenance therapy of lung cancer mainly due to its low toxicity or adverse effects, less cost, and better improvement in clinical outcomes [29, 30]. TCM scholars deem that Chinese herbs change the tumor microenvironment, such that tumor cells become dormant or difficult to survive in the “soil”. The adverse effects or costs of Chinese herbs and anti-inflammatory drugs are far lower than the targeted drugs or chemotherapy drugs. However, no previous experimental study has investigated the effect of Chinese herbs in combination with anti-inflammatory drugs for preventing tumor metastasis.

FLP ointment inhibits lung cancer invasion by regulating tumor inflammatory microenvironment through the NF-κB signaling pathway [31]. Celecoxib improves tumor microenvironment through inhibiting the Cox-2 pathway. Although FLP ointment has shown to be effective in treating lung cancer in vivo, the mechanisms of action remain unclear. Whether FLP ointment in combination with celecoxib exerts synergistic effects in improvement of tumor inflammatory microenvironment or prevents progression and metastasis is still poorly understood. The objective of the current study is to investigate the effect of FLP alone or in combination with celecoxib on tumor growth in Lewis lung xenograft model and Cox-2 expression in lung cancer specimens. Furthermore, we also sought to examine the effect of FLP on Cox-2, and other metastasis-related protein expression, in normal lung tissues.

## Methods

### Drugs and antibodies

FLP ointment (No. Z20063236) was prepared by Pharmaceutical preparation center of Guang’anmen Hospital, China Academy of Chinese Medical Sciences. Celecoxib (No. H20120355) was purchased from Pfizer Pharmaceuticals LLC (Dalian, China). Antibodies against Cox-2, E-Cadherin, Vimentin, platelet-derived growth factor receptors (PDGFR) β, β-actin, GAPDH, anti-rabbit IgG, and horseradish peroxidase (HRP)-linked antibody was purchased from Cell Signaling Technology (Cambridge, MA, USA). Vascular endothelial growth factor (VEGF), N-Cadherin, microsomal Prostaglandin E synthase-1 (mPGES-1), matrix metalloproteinases (MMP)-2, and MMP-9 antibodies were purchased from Abcam (Cambridge, MA, USA). Biotinylated secondary antibodies were purchased from Beijing Xiya Jinqiao Biological Technology Co. Ltd.

### Preparation of FLP ointment

The herbs used in FLP ointment are the roots of *Astragalus membranaceus (Fisch.) Bge.var.mongholicus (Bge.) Hsiao* (Huang-Qi), *Panax quinquefolium L.* (Xi-Yang-Shen), *Ophiopogon japonicas (Thunb.) Ker*-*Gawl.* (Mai-Dong), *Glehnia littoralis Fr. Schmidt ex Miq.* (Bei-Sha-Shen), *Agrimonia pilosa Ledeb.* (Xian-He-Cao), *Polygonum bistorta L.* (Quan-Shen), *Patrinia villosa (Thunb.) Juss.* (Bai-Jiang-Cao), *Panax notoginseng (Burk.) F.H. Chen* (San-Qi), *Fritillaria cirrhosa D.Don* (Chuan-Bei-Mu), *Glycyrrhiza uralensis Fisch.* (Gan-Cao), *Cordycrps sinensis(Berk.) Sacc.* (Dong-Cong-Xia-Cao), and the fruits of *Prunus persica (L.) Batsch* (Tao-Ren) and *Prunus armeniaca L. var ansu Maxim.* (Xing-Ren). All herbs were provided by the Guang’anmen Hospital and decocted twice with eightfold volume of distilled water for 1 h. The decoction were collected, filtered, merged and concentrated to 2 g/mL (equivalent to crude herb materials), and stored at 4 °C for oral use.

### Animals

Specific pathogen free 6–8-weeks-old male C57BL/6 mice weighing 20 ± 2 g were purchased from Vital River Company (Beijing, China). All the mice were kept under a temperature and humidity-controlled animal facility with a 12-h light/dark cycle. Mice were had free access to feed pellets and tap water. All the experiments were carried out in accordance with the guidelines for animal experiments of China Academy of Chinese Medical Sciences (IACU numbers: SYXK [Beijing], 2012-0034).

### Cell culture

LL/2-luc-M38 cells were kindly provided by the cancer institute of Guang’anmen Hospital, Chinese Academy of Science. Tumor cells were cultured in Dulbecco’s modification of Eagle’s medium Dulbecco (DMEM) medium containing 10 % fetal bovine serum, 100 U/ml penicillin, and 100 mg/ml streptomycin in a cell culture incubator at 37 °C under 5 % CO_2_. Cells were collected at the logarithmic phase of growth by treatment with 0.25 % trypsin for 1 min, then the cell concentration was adjusted to 1 × 10^6^ with phosphate buffer saline (PBS). Trypan Blue exclusion test indicated the number of living cells was greater than 95 %.

### Induction of Lewis lung cancer and intervention

After 3-day acclimation, a subcutaneous injection of LL/2-luc-M38 cells 5 × 10^5^ suspended in 0.2 mL PBS was implanted into the right flank of each C57BL/6 mouse. Starting the next day, forty mice were randomly divided equally into four groups: the vehicle control group (received the normal saline by gavage), FLP ointment treated group (received 12 g/kg/day FLP by gavage), celecoxib treated group (received celecoxib at doses of 3200 ppm/day in the diet from date of implant until end of study [32]), and FLP ointment plus celecoxib group (received a combination of celecoxib and FLP treatments described above). The mice were treated up to 28 days, and some of the mice were sacrificed by cervical decapitation under ether anesthesia at day 14, day 21, and day 28, and tumor specimens and normal lung tissues were harvested and weighed. Part of specimens were exposed to 4 % paraformaldehyde fixation for hematoxylin-eosin (HE) staining and immunohistochemistry study, and the remaining tissues were kept in −80 °C for Western blot analysis.

### In vivo bioluminescent imaging

In order to observe the dynamic change of inhibitory effect of FLP on tumor growth, we compared in vivo tumor imaging with the volume of tumor from the sacrificed mice. Imaging was performed at day 7, 14, 21, and 28 after treatment (N = 6 for each group). D-luciferin (Biotium, Hayward, CA, USA) was dissolved in 15 g/L PBS and injected intraperitoneally at a dose of 10 μL/g body weight before imaging. Imaging was performed 40 min later, and then the mice were anesthetized under gas-induced anesthesia, and placed in the imaging chamber. Bioluminescent images were acquired by a cryogenically cooled charge-coupled device camera (IVIS Lumina Imaging System, Caliper Life Sciences Inc). A region of interest (ROI) was drawn from each tumor location and the signal was calculated based on the number of photons from mouse body surface area (per/s/cm^2^/sr). Red signal represents the high intensity, and purple signal represents the low intensity. Finally, the collected data were analyzed by statistical analysis.

### Tumor inhibition rate

Mice were sacrificed by cervical dislocation at day 14, day 21, and day 28, and then the tumor tissue were harvested and weighed. Tumor inhibition rate was calculated as follows: Tumor inhibition rate = (1 − average weight of tumors in treatment group/average weight of tumors in control group) × 100 %.

### Histological staining and immunohistochemistry

Tumor specimens and normal lung tissues were harvested and immersed into 4 % paraformaldehyde fixation for 20 h. All the paraffin embedded samples were cut into 5 μm sections for HE staining, according to standard protocols. For immunohistochemistry study, each section was dehydrated through a graded series of ethanol solutions. Antigen retrieval was performed by incubating the specimens with ethylene diamine tetraacetic acid (EDTA) in a microwave oven for 150 s. After rinsing with PBS for 3 times, these sections were treated with 3 % hydrogen peroxide for 10 min to inactivate endogenous peroxides. After blocking with 1 % goat serum in PBS for 10 min at room temperature, sections were incubated with primary anti-Cox-2 (1:200 dilution), anti- mPGES-1 (1:100 dilution), anti- E-Cadherin (1:300 dilution), anti-N-Cadherin (1:300 dilution), anti-Vimentin (1:500 dilution), anti-VEGF (1:50), anti-PDGFRβ (1:50), anti-MMP-2 (1:50), or anti-MMP-9 (1:200) antibodies overnight at 4 °C. Sections were then incubated with horseradish peroxidase (HRP)-conjugated secondary antibody for 20 min at 37 °C followed by incubation with diaminobenidine (DAB) for 3 min. The slides were stained with hematoxylin, differentiated with 1 % hydrochloric acid alcohol, and stained blue with 1 % ammonia water. Finally stained tissues were analyzed by light microscopy. Negative controls were incubated with PBS instead of primary antibodies.

### Western blot analysis

Proteins were isolated from tumor specimens and normal lung tissues and the expression levels were measured using Western blot analysis. Briefly, 100 mg of the tumor specimens or normal lung tissues were minced on ice, washed 2 times with the cold PBS, and homogenized 20–40 times in 1 mL mixture of radio-immunoprecipitation assay (RIPA) buffer with protease inhibitor (Applygen Technologies Inc, Beijing, China). After an ice bath for 10 min, homogenized samples were centrifuged at 1000 rpm for another 10 min, and the supernatants were collected. Protein concentration was calculated using standard bovine serum albumin (BSA) curve. Approximately 30 μg of total proteins were separated by sodium dodecyl sulfate–polyacrylamide gel electrophoresis and electrophoretically transferred to nitrocellulose membranes. The membranes were blocked with 5 % milk in Tris-buffered saline (TBS) for 2 h at room temperature, followed by incubation with anti-Cox-2 (1:1000 dilution), anti- mPGES-1 (1:500 dilution), anti-E-Cadherin (1:1000 dilution), anti-N-Cadherin (1:1000 dilution), anti-Vimentin (1:1000 dilution), anti-VEGF (1:500), anti-PDGFRβ (1:1000), anti-MMP-2 (1:300), anti-MMP-9 (1:1000), anti-β-actin (1:1000), or anti-GAPDH (1:1000) antibodies. The membranes were incubated on a shaker overnight at 4 °C and then washed three times before incubating with the HRP-conjugated secondary antibodies at a dilution of 1:2000 for 1 h at room temperature. The bands were visualized by adding ECL detection reagent (Amersham Life Science, Piscataway, NJ, USA). The membranes were exposed to X-ray film and photographed by BIO-RAD ChemiDocXRS gel imaging system. The pictures were exported by using Quantity One software. The intensities of the protein bands for target protein were quantified relative to β-actin or GAPDH bands from the same sample using image analysis software ImageProPlus 4.5 (Diagnostic Instruments,USA).

### Statistical analysis

All data are presented as mean ± standard deviation (SD), while categorical variables are expressed as observed frequencies. One-way repeated-measures analysis of variance (ANOVA) and multiple comparisons tests were applied with Prism 6.0. All statistical tests were two-sided test, and *p*-values <0.05 were considered to be statistically significant.

## Results

FLP ointment treatment exerted the best tumor growth inhibition rate at day 21 in Lewis lung cancer xenograft mice, and FLP in combination with celecoxib enhanced the antitumor effects.

**Fig. 1:**
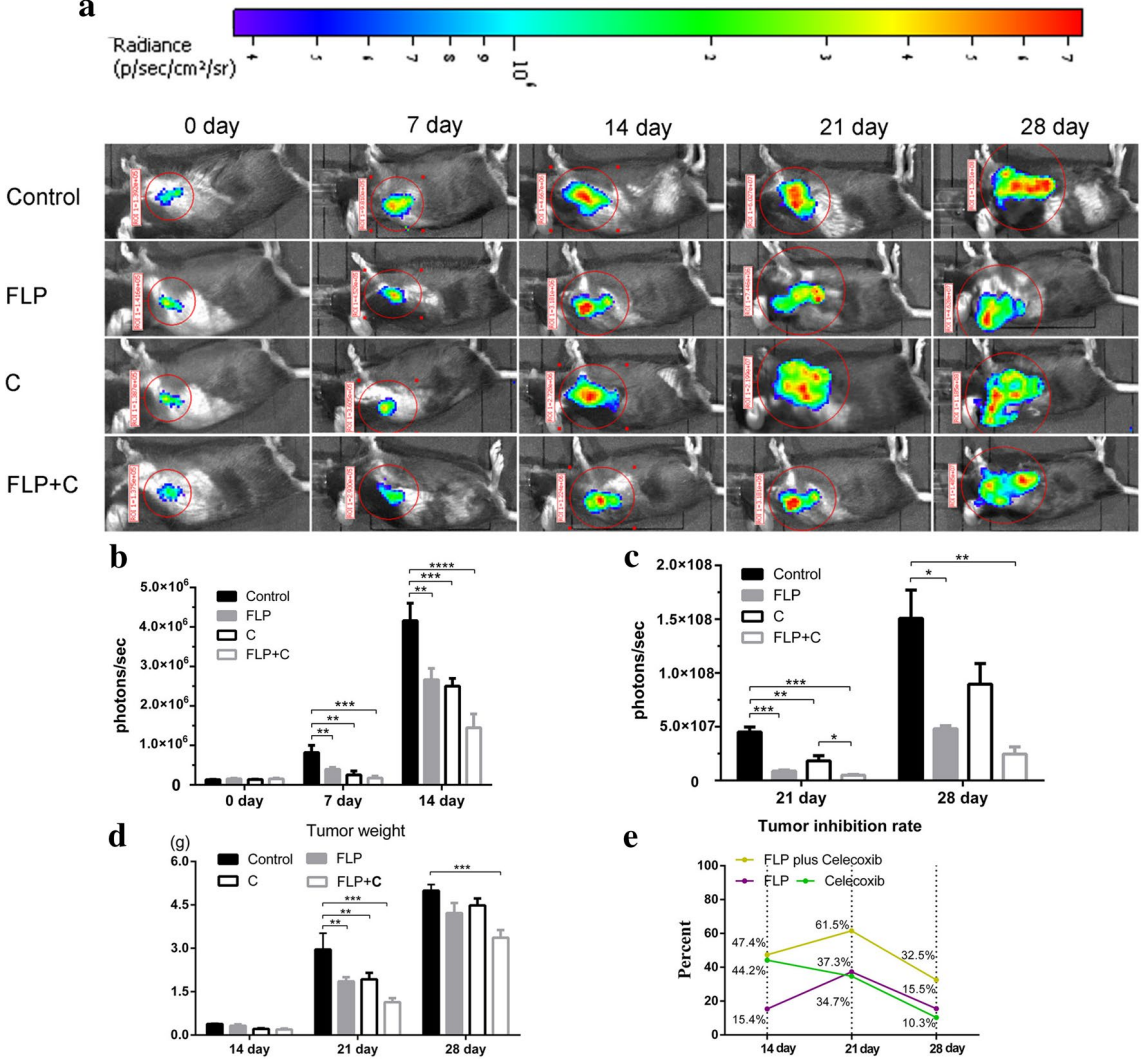
Effects of FLP ointment on tumor growth in Lewis lung carcinoma xenograft mice. 5 × 10^5^ LL/2-luc-M38 cells were implanted subcutaneously in C57BL/6 mouse. **a** Dynamic change of tumor growth measured by vivo bioluminescent signal; **b** The magnitude of tumor growth in vivo bioluminescent signal (p/s/cm^2^/s) at day 0, day 7, and day 14. **c** The magnitude of tumor growth in vivo bioluminescent signal (p/s/cm^2^/s) at day 21 and day 28. **d** Tumor weight of the sacrificed mice at day 14, day 21, and day 28. **e** Tumor inhibition rate was calculated by (1 − average weight of tumors in treatment group/average weight of tumors in control group) × 100 %. Values were expressed as mean ± SD. **p < 0.01, *p < 0.05, ***p < 0.001, and ****p < 0.0001. *FLP* Fei-Liu-Ping ointment group, *C* celecoxib group, *FLP* *+* *C* Fei-Liu-Ping ointment plus celecoxib group. FLP ointment inhibited the tumor growth, while in combination with celecoxib exerted more significant anti-tumor growth effect at day 21 and day 28, and these findings were consistent with in vivo imaging results

Lewis lung cancer xenograft mice were used to evaluate the tumor growth inhibition effects of FLP, celecoxib, and FLP in combination with celecoxib. At five time points (day 0, day 7, day 14, day 21, and day 28) we performed in vivo bioluminescence imaging and collected tumor from mice sacrificed by cervical dislocation at 3 time points (day 14, day 21, and, day 28). The number of photons from mouse body surface area (Fig. 1a–c) and the tumor weight (Fig. 1d, e) were compared among each treatment group. In vivo bioluminescence imaging experiments showed that FLP ointment treatment significantly reduced the number of photons from the mouse body surface area (*p* < 0.001). Moreover, FLP ointment treatment significantly reduced tumor weight at day 21 compared with the control group (*p* < 0.01) and exhibited the highest tumor growth inhibition rate (37.3 %). These findings were consistent with in vivo imaging results. Specially, FLP ointment in combination with celecoxib was more effective in reducing per/s/cm^2^/sr and increasing the tumor growth inhibition rate compared with the FLP ointment alone, or celecoxib alone.

**Fig. 2:**
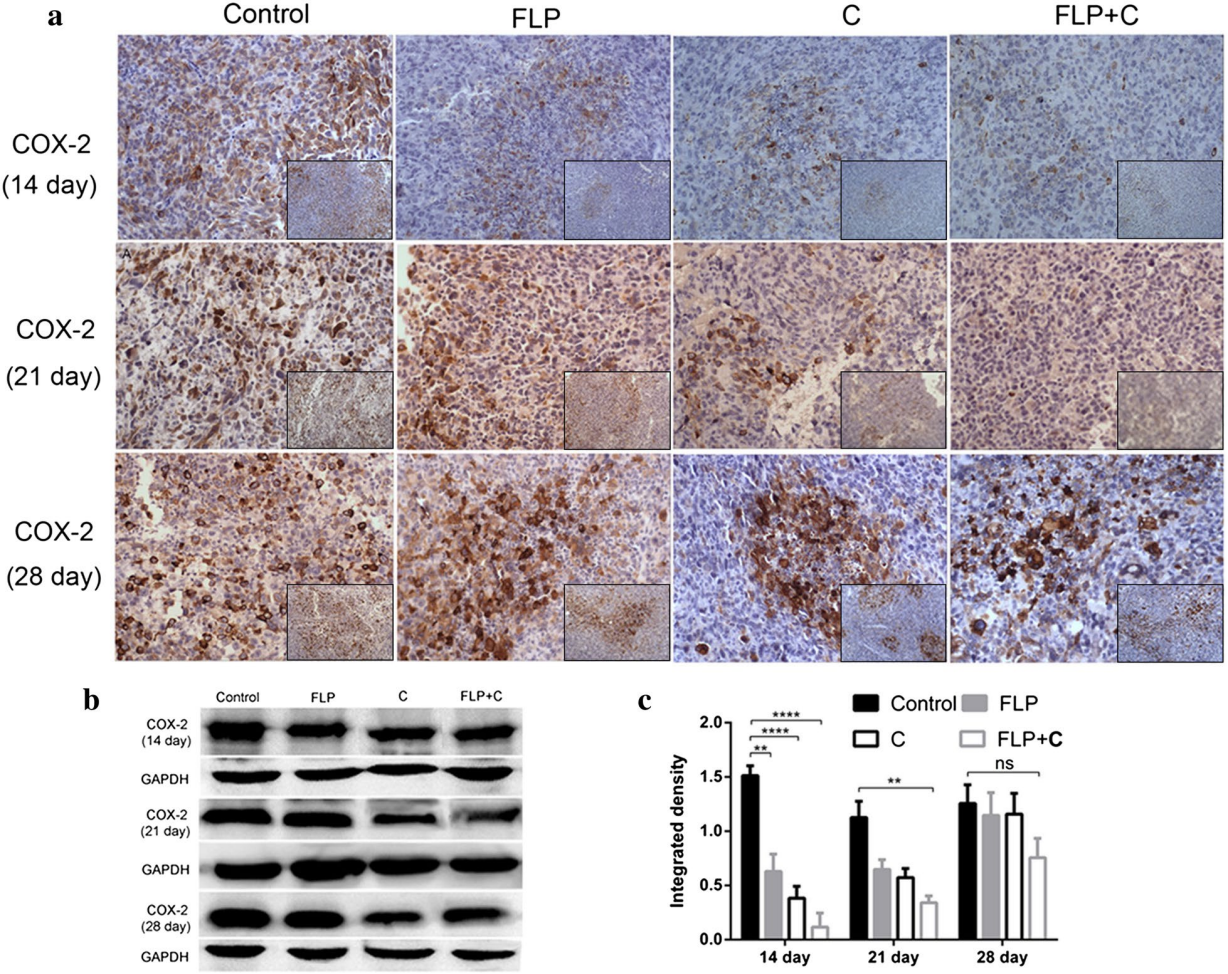
Effects of FLP ointment on Cox-2 expression in the tumor specimens. **a** Immunohistochemical analysis of Cox-2 expression (×200 magnification and ×100 magnification). The *brown* or *brown yellow* represented positive Cox-2 protein. **b** A representative band of Cox-2 protein expression by Western blot analysis. **c** Relative protein level of Cox-2 (*n* = 3). Values were expressed as mean ± SD. **p < 0.01, *p < 0.05, ***p < 0.001, and ****p < 0.0001. *FLP* Fei-Liu-Ping ointment group, *C* celecoxib group, *FLP* *+* *C* Fei-Liu-Ping ointment plus celecoxib group. FLP ointment significantly inhibited the Cox-2 expression in tumor specimens at the initial stage without exerting time-dependent inhibitory effect. FLP ointment in combination with celecoxib did not show obvious advantage in inhibiting Cox-2 expression

FLP ointment significantly inhibited the expression of Cox-2 in the tumor specimens at day 14, while in combination with celecoxib did not exert obvious advantages in inhibiting Cox-2 expression. Cox-2 is one of the key factors in maintaining the tumor inflammatory microenvironment. Cox-2 over-expression was found in lung cancer and was closely related to the prognosis of lung cancer patients [23]. We measured Cox-2 expression from the tumor specimens and normal lung tissue by immunohistochemistry and Western blot analysis at day 14, day 21, and day 28. The results showed that there was no obvious change in Cox-2 expression in the control group over time. However, FLP, celecoxib, or FLP in combination with celecoxib treatment inhibited Cox-2 expression compared with the control group at day 14 (Fig. 2). Interestingly, there was no significant difference in Cox-2 expression between celecoxib alone and FLP in combination with celecoxib at day 14, whereas only FLP in combination with celecoxib treatment significantly inhibited Cox-2 expression (*p* < 0.01) at day 21 compared with the control group. On day 28, there was no significant difference in Cox-2 expression between treatment groups and the control group.

### Large number of inflammatory cell infiltrations were observed in the alveolar septum of Lewis lung cancer mice before and after metastasis

**Fig. 3:**
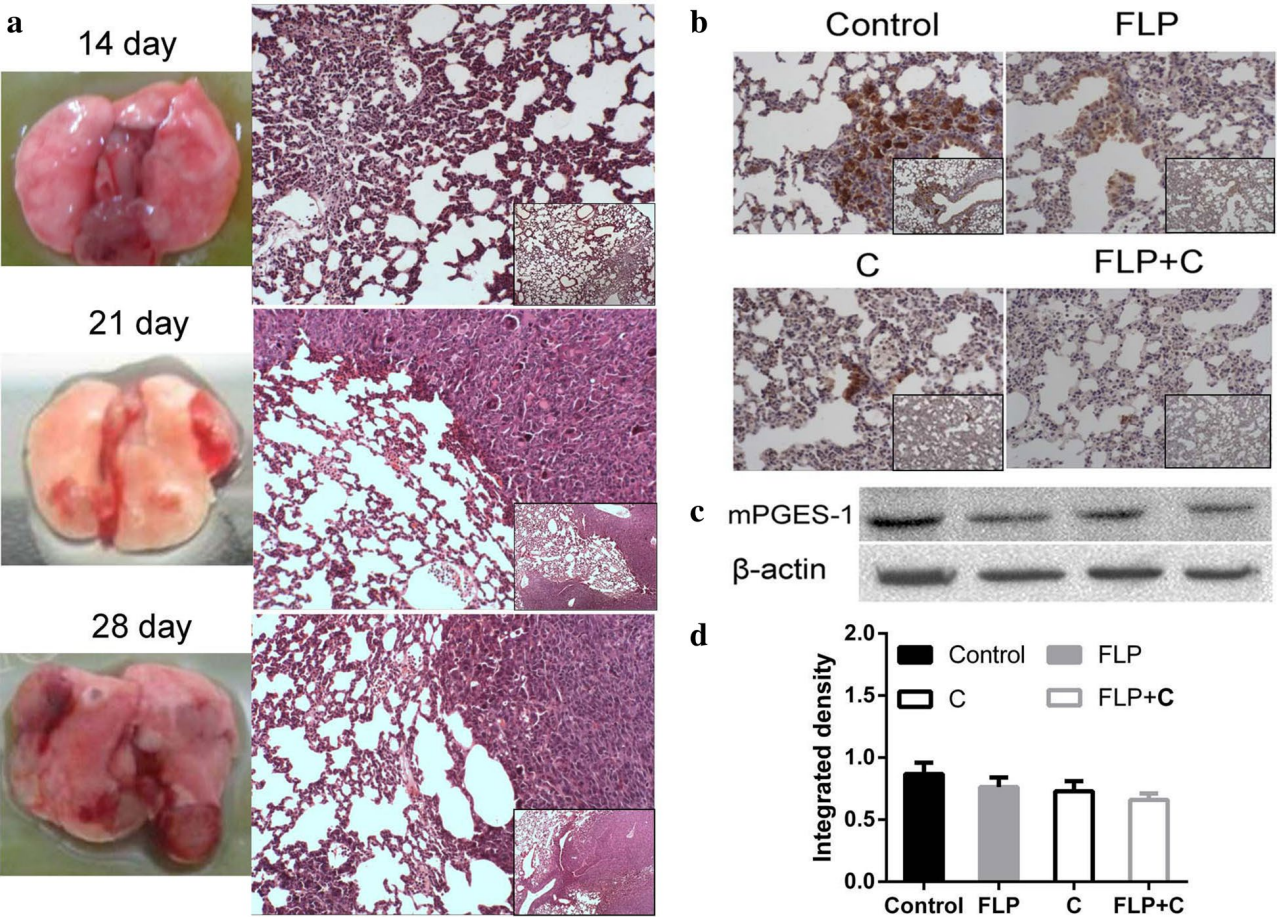
Effects of FLP ointment on histological changes and mPGES-1 expression in the lung tissues. **a** Histological analysis of the lung tissues in Lewis lung carcinoma xenograft mice. Large number of inflammatory cell infiltration was found in the alveolar septum around the metastasis location in the control group, while inflammatory cell infiltration was alleviated in the treatment group. **b** Immunohistochemical analysis of mPGES-1 expression in lung tissues (×200 magnification and ×100 magnification). The *brown* or *brown yellow* represented positive mPGES-1 protein. **c** A representative band of mPGES-1 protein expression by Western blot analysis. **d** Relative protein level of mPGES-1 (*n* = 3). Values were expressed as mean ± SD. **p < 0.01, *p < 0.05, ***p < 0.001, and ****p < 0.0001. *FLP* Fei-Liu-Ping ointment group, *C* celecoxib group, *FLP* *+* *C* Fei-Liu-Ping ointment plus celecoxib group. FLP ointment in combination with celecoxib exerted a synergistic effect in inhibiting mPGES-1 expression

At day 28, we sacrificed mice and collected lung tissues to observe the rate of lung metastasis. The lung metastasis rate was 80, 50, 60, and 30 % and the total numbers of metastatic nodules were 52, 20, 29, and 8 in the control group, FLP group, celecoxib group, and FLP with celecoxib group, respectively. In order to examine the inflammatory changes in lung tissues, we observed the changes of lung tissues before and after metastasis in the control group. The histological images indicated that a large number of inflammatory cell infiltrations were observed in the alveolar septum before the metastasis formation (Fig. 3a-day 14), and after the formation of metastases (Fig. 3a-day 21). With the progression of tumor metastases, large numbers of new inflammatory cell infiltrations in the alveolar septum of the location around the metastases were found (Fig. 3a-day 28). This has raised questions as follows: does the Cox-2 expression change along with the time sequence in the lung metastatic microenvironment? Can anti-tumor metastasis effect of FLP ointment, celecoxib, or FLP ointment in combination with celecoxib regulate tumor metastasis inflammatory microenvironment by inhibiting Cox-2 expression? To better understand these questions, we conducted the following experiments.

### FLP ointment in combination with celecoxib synergistically inhibited Cox-2 expression in the tumor metastasis microenvironment in a time-dependent manner

**Fig. 4:**
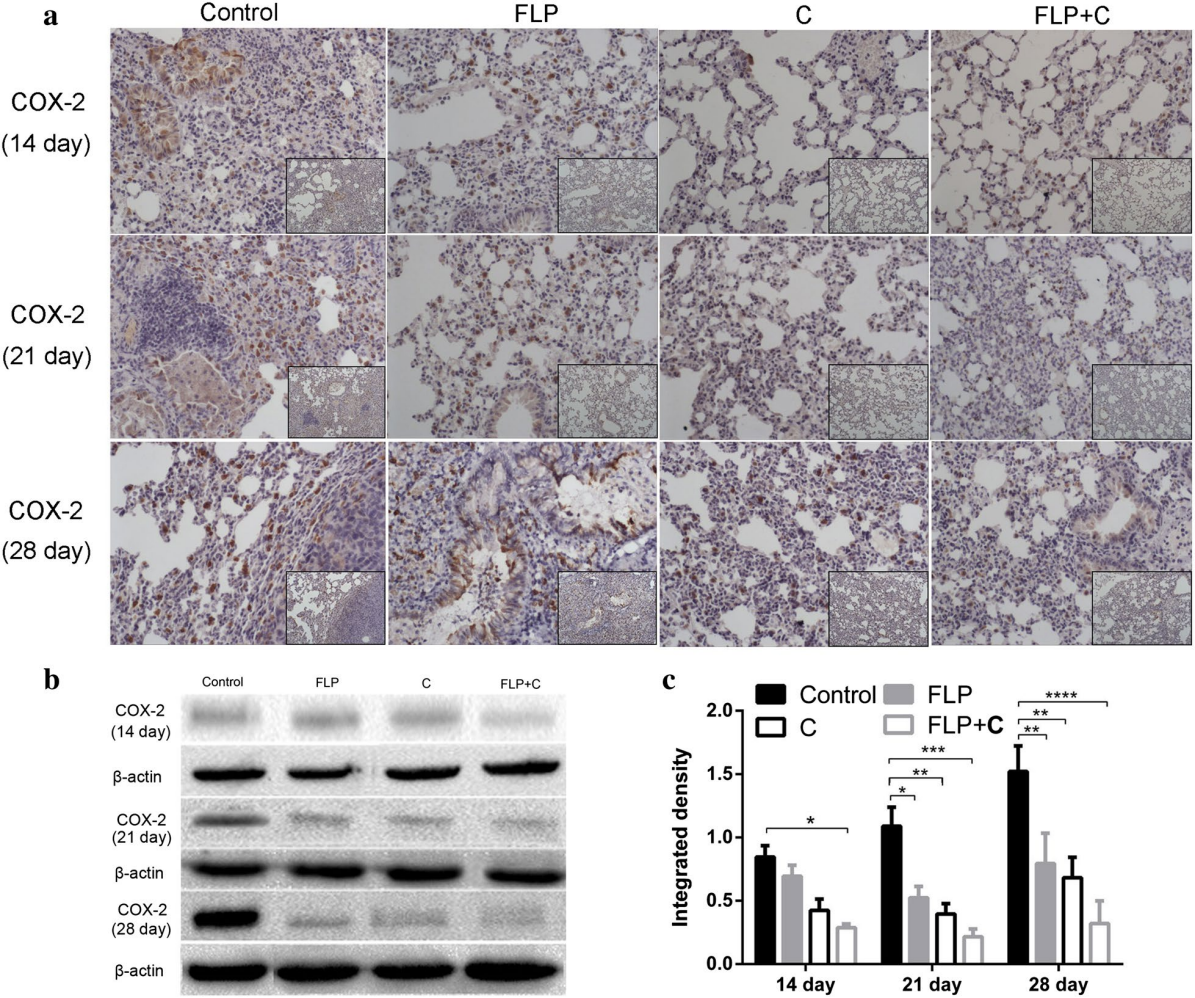
Effects of FLP ointment on Cox-2 expression in the lung tissues. **a** Immunohistochemical analysis of Cox-2 expression (×200 magnification and ×100 magnification). The *brown* or *brown yellow* represented positive Cox-2 protein. **b** A representative band of Cox-2 protein expression by Western blot analysis. **c** Relative protein level of Cox-2 (*n* = 3). Values were expressed as mean ± SD. **p < 0.01, *p < 0.05, ***p < 0.001, and ****p < 0.0001. *FLP* Fei-Liu-Ping ointment group, *C* celecoxib group, *FLP* *+* *C* Fei-Liu-Ping ointment plus celecoxib group. FLP ointment in combination with celecoxib synergistically inhibited Cox-2 protein expression in the lung metastatic inflammatory microenvironment

Cox-2 expression is known to contribute to tumor metastasis. We measured Cox-2 protein in the lung tissues by immunohistochemistry and Western blot analysis at day 14, day 21, and day 28. The results showed that Cox-2 expression was increased in a time-dependent manner in the control group. FLP ointment markedly inhibited Cox-2 expression at day 21 compared with the control group (*p* < 0.05), and the inhibitory effect was increased at day 28 (*p* < 0.01). Celecoxib significantly inhibited Cox-2 expression at day 21 and day 28 (*p* < 0.01). FLP ointment in combination with celecoxib significantly inhibited Cox-2 expression over time, and the inhibitory effect was increased in a time-dependent manner (Fig. 4). In addition, inflammatory cell infiltration was attenuated to some extent in the treatment group. Since FLP ointment alone or FLP ointment in combination with celecoxib inhibited Cox-2 expression in a time-dependent manner, we determined that the strongest inhibition occurred at day 28. We further investigated the changes of its downstream targets and metastasis related mPGES-1, epithelial to mesenchymal transition (EMT), ECM, and angiogenesis-related protein expression.

### FLP ointment in combination with celecoxib reduced mPGES-1 expression in the lung metastasis microenvironment

Prostaglandin E2 (PGE2) can promote angiogenesis and tumor metastasis, attenuate cell apoptosis, and inhibit tumor immunity in various tumors [33]. PGE2 is derived from the enzymatic release of arachidonic acid, which is metabolized by Cox and specific PGE syntheses. The mPGES-1, a subtype of PGE syntheses, is an inducible enzyme that functions downstream of Cox- 2 in the PGE2 -biosynthesis pathway and often over-expressed in non-small cell lung cancer [34]. Overexpression of mPGES-1 contributes to tumor growth, invasion, and metastasis [35]. As shown in Fig. 3b, mPGES-1 over-expression was identified in lung tissues of Lewis lung cancer mice. FLP ointment in combination with celecoxib was more effective than FLP ointment or celecoxib alone in inhibiting mPGES-1 expression. Immunohistochemistry image indicated that FLP ointment more effectively inhibited mPGES-1 expression than celecoxib alone, while Western blot results showed that there was no statistically significant difference in mPGES-1 protein expression between the treatment and control groups (*p* > 0.05).

### FLP ointment in combination with celecoxib inhibited VEGF and PDGFRβ expression

**Fig. 5:**
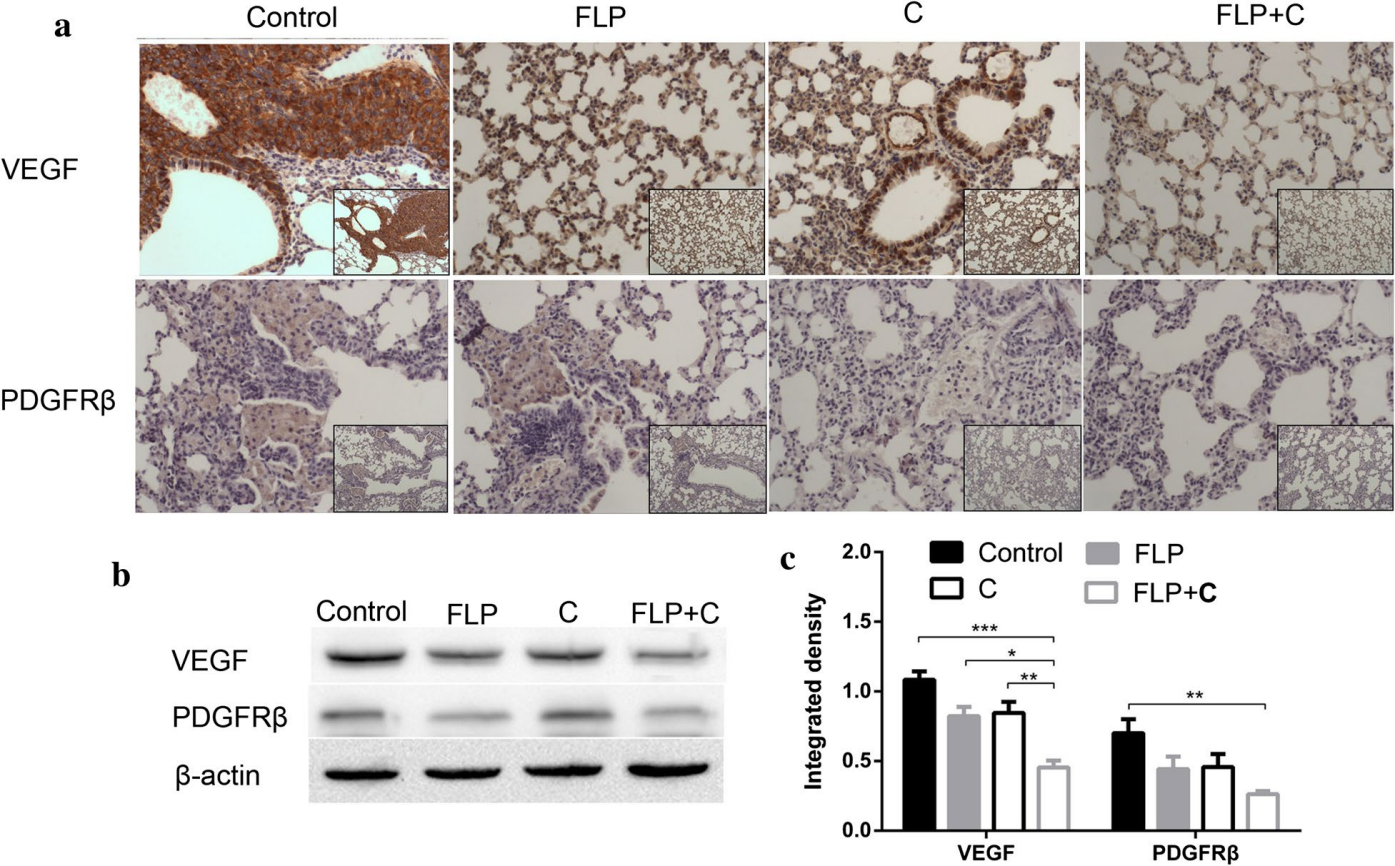
Effects of FLP ointment on VEGF and PDGFRβ expression in the lung tissues. **a** Immunohistochemical analysis of VEGF and PDGFRβ expression (×200 magnification and ×100 magnification). The *brown* or *brown yellow* represented positive VEGF and PDGFRβ protein. **b** A representative band of VEGF and PDGFRβ protein expression by Western blot analysis. **c** Relative protein level of VEGF and PDGFRβ (*n* = 3). Values were expressed as mean ± SD. **p < 0.01, *p < 0.05, ***p < 0.001, and ****p < 0.0001. *FLP* Fei-Liu-Ping ointment group, *C* celecoxib group, *FLP* *+* *C* Fei-Liu-Ping ointment plus celecoxib group. FLP ointment in combination with celecoxib inhibited VEGF and PDGFRβ protein expression in the lung metastatic inflammatory microenvironment

Angiogenesis is integral for tumor development, providing nutrients necessary for cell growth. Angiogenesis is required for the initiation of metastasis and plays an important role during initiation and end process of tumor metastasis. VEGF is the strongest angiogenesis stimulating factor [36]. PDGFRβ expression in endothelial cells regulates VEGF transcription and secretion. The PDGF/PDGF receptor pathway is located on pericytes of the tumor stroma, and plays a pivotal role in angiogenesis and vessel maturation [37, 38]. As shown in Fig. 5, FLP ointment significantly inhibited VEGF protein expression (*p* < 0.05). FLP ointment in combination with celecoxib exhibited the strongest inhibitory effect on VEGF protein expression (*p* < 0.001). Furthermore, FLP ointment combined with celecoxib significantly inhibited PDGFRβ protein expression (*p* < 0.01).

### FLP ointment in combination with celecoxib regulated EMT-related proteins expression in the lung tissues of Lewis lung cancer mice

**Fig. 6:**
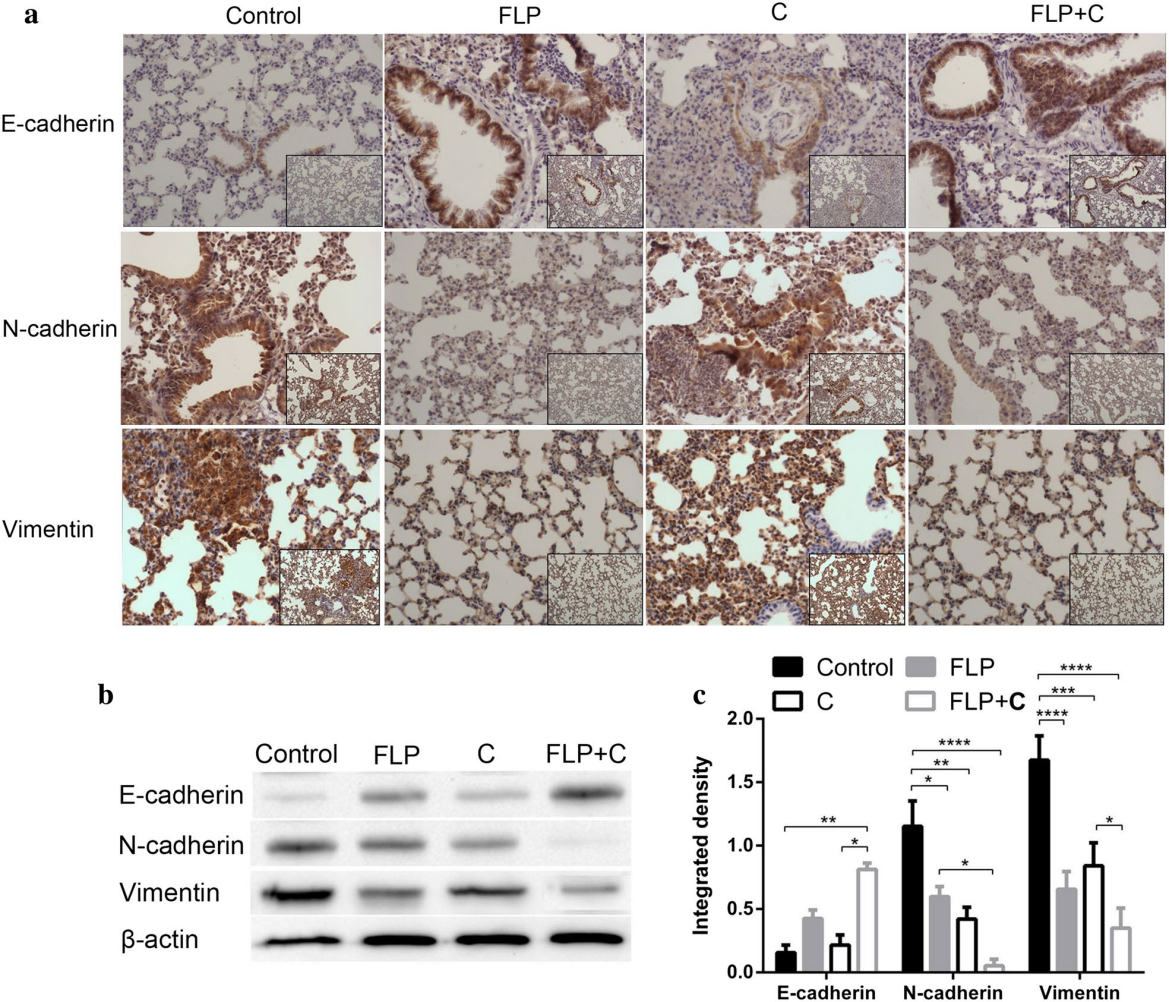
Effects of FLP ointment on E-cadherin, N-cadherin, and Vimentin expression in the lung tissues. **a** Immunohistochemical analysis of E-cadherin, N-cadherin, and Vimentin expression (×200 magnification and ×100 magnification). The *brown* or *brown yellow* represented positive E-cadherin, N-cadherin, and Vimentin protein. **b** A representative band of E-cadherin, N-cadherin, and Vimentin protein expression by Western blot analysis. **c** Relative protein level of E-cadherin, N-cadherin, and Vimentin (*n* = 3). Values were expressed as mean ± SD. **p < 0.01, *p < 0.05, ***p < 0.001, and ****p < 0.0001. *FLP* Fei-Liu-Ping ointment group, *C* celecoxib group, *FLP* *+* *C* Fei-Liu-Ping ointment plus celecoxib group. FLP ointment in combination with celecoxib synergistically inhibited EMT-related E-cadherin, N-cadherin, and Vimentin protein expression in the lung metastatic inflammatory microenvironment

EMT is known to be involved in the invasion and metastasis of malignant tumor cells [39]. EMT is defined as switching of polarized epithelial cells to a migratory fibroblastoid phenotype. EMT refers the morphology from epithelial to mesenchymal transition and achieves the migratory capacity, which might be a bridge between inflammation and tumor [40, 41]. The characteristics of EMT include the loss of EMT-related proteins expression, such as E-cadherin and keratin, and the up-regulation of interstitial related proteins expression, including N-cadherin, Vimentin, and Fibronectin. We measured E-cadherin, N-cadherin, and Vimentin expression in the lung tissue of Lewis lung cancer mice by immunohistochemistry and Western blot analysis. As shown in Fig. 6, E-cadherin expression was reduced, and N-cadherin and Vimentin expression was enhanced in the control group. FLP ointment increased E-cadherin expression, but significantly reduced N-cadherin and Vimentin expression.

### FLP ointment in combination with celecoxib effectively reduced ECM-related proteins expression

**Fig. 7:**
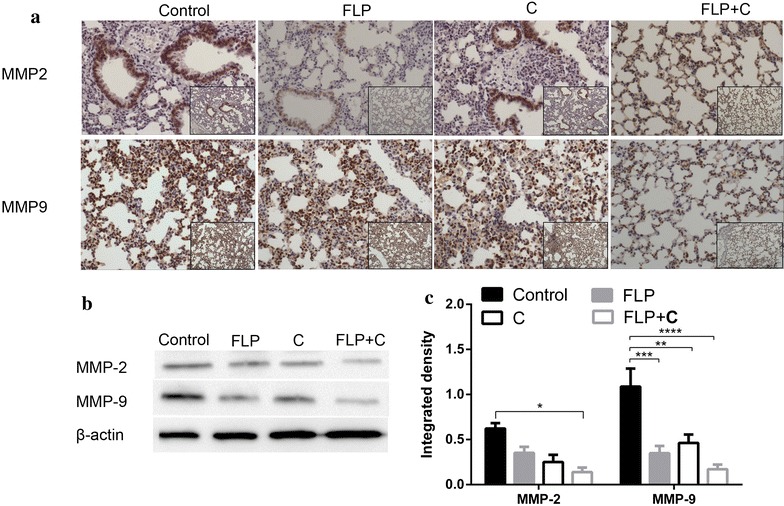
Effects of FLP ointment on MMP-9 and MMP-2 expression in the lung tissues. **a** Immunohistochemical analysis of MMP-9 and MMP-2 expression (×200 magnification and ×100 magnification). The *brown* or *brown yellow* represented positive Cox-2 protein. **b** A representative band of MMP-9 and MMP-2 protein expression by Western blot analysis. **c** Relative protein level of MMP-9 and MMP-2 (*n* = 3). Values were expressed as mean ± SD. **p < 0.01, *p < 0.05, ***p < 0.001, and ****p < 0.0001. *FLP* Fei-Liu-Ping ointment group, *C* celecoxib group, *FLP* *+* *C* Fei-Liu-Ping ointment plus celecoxib group. FLP ointment in combination with celecoxib synergistically inhibited ECM-related MMP-9 and MMP-2 protein expression in the lung metastatic inflammatory microenvironment

ECM is an important tissue barrier for tumor metastasis. Deregulation of the ECM and basement membrane is the basic prerequisite for the cancer cell invasion and angiogenesis [34, 42]. One of the critical factors for tumor invasion is a large production of MMPs that principally participates in the degradation of basement membranes and ECM [43]. Type-IV collagen is a major constituent of the basement membrane. The major MMPs involved in tumor angiogenesis are MMP-2 and MMP-9 [44]. MMP-2 and MMP-9 play critical roles in the degradation of type-IV collagen [45]. Moreover, MMP-9 mainly regulates the bioavailability of VEGF, the most potent inducer of tumor angiogenesis [46]. As shown in Fig. 7, FLP ointment inhibited the MMP-2 expression and MMP-9 (*p* < 0.001). FLP ointment in combination with celecoxib further inhibited the expression of MMP2 and MMP9.

## Discussion

This study investigated the effect of FLP on the regulation of tumor metastatic inflammatory microenvironment. FLP ointment inhibited Cox-2 expression of the tumor specimens. More importantly, it also inhibited Cox-2 expression of the normal lung tissues in the tumor metastatic inflammatory microenvironment in a time-dependent manner. Our study demonstrated for the first time that FLP ointment in combination with celecoxib adjunctly regulates Cox-2 mediated metastasis-related proteins expression in the microenvironment. Thus, integrated therapy provided better efficacy in the prevention of lung metastasis in the Lewis lung carcinoma xenograft mouse model.

**Fig. 8:**
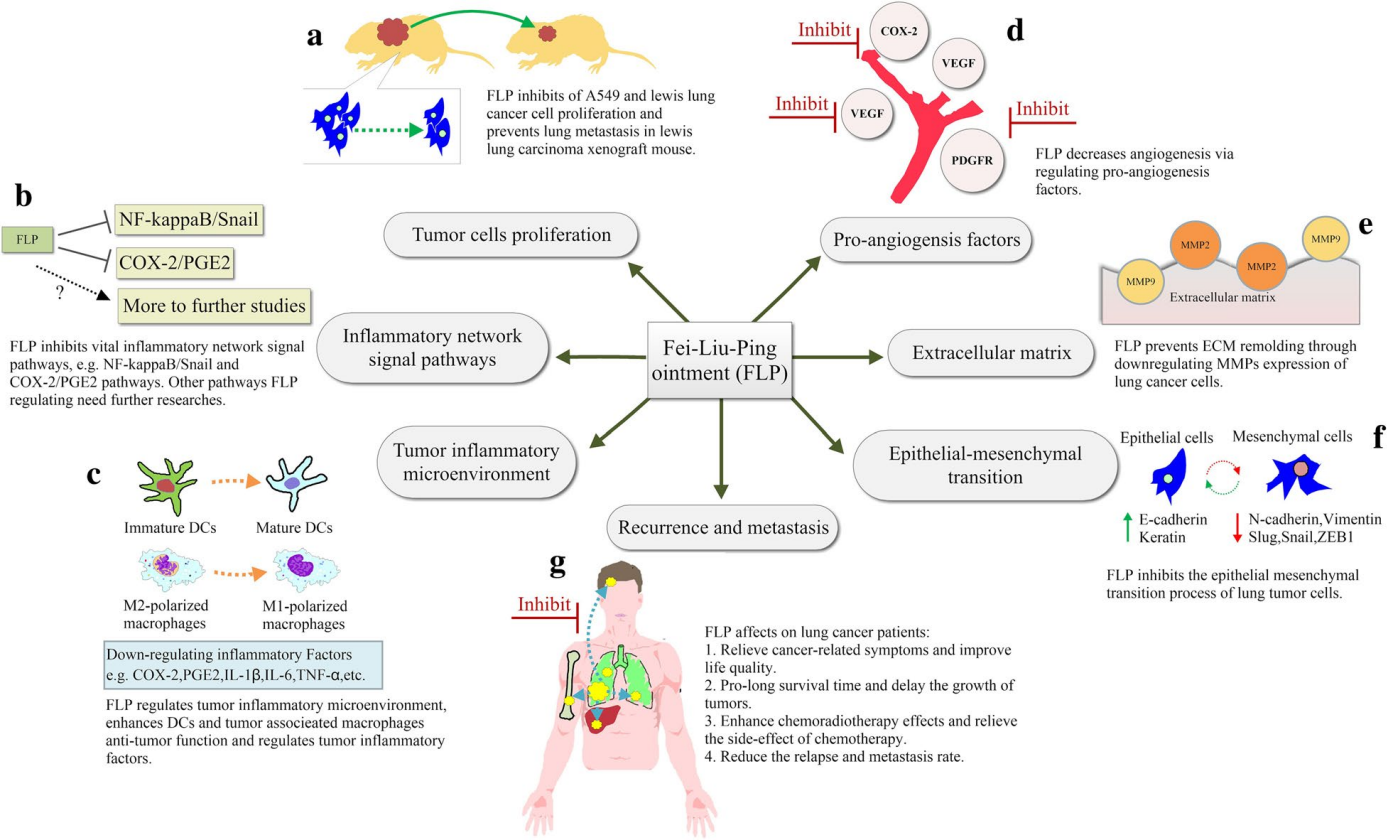
Potential mechanisms of FLP ointment on the lung cancer treatment and prevention. **a** FLP ointment can inhibit the proliferation of A549 cells. **b** Possible inflammatory network signal pathways of FLP ointment. **c** Previous and the current experiment demonstrated that FLP ointment inhibited metastasis through the regulation of tumor inflammatory microenvironment. **d**–**f** Previous and the current experiment showed that FLP ointment inhibited angiogenesis, prevented extracellular matrix remodeling, and regulated epithelial-mesenchymal transition. **g** Clinical trials confirmed the efficacy of FLP ointment

FLP ointment has been used to treat lung cancer and has been suggested to prevent lung cancer metastasis or recurrence for many years in China [47]. FLP ointment has been developed based on the Differentiation of Syndrome of TCM theory for lung cancer or lung metastasis patients with deficiency of both Qi and Yin. FLP has potential to improve the quality of life, inhibit tumor growth, and prolong survival of lung cancer patients. Our previous study [31] showed that FLP promotes dendritic cell maturation and regulates tumor inflammatory microenvironment in vitro, and inhibits tumor growth and invasion through regulating NF- kappa B pathway in tumors of the Lewis lung carcinoma xenograft mouse model in vivo. Moreover, FLP also reduces serum TNF-α, IL-1β, and IL-6 level in a Lewis lung carcinoma xenograft mouse. The potential mechanism of action of FLP ointment on the lung cancer treatment and prevention is presented in Fig. 8. The current study indicated that the tumor inhibition rate was 37.3 % at day 21, and this finding was consistent with the absolute number of photons from mouse body surface area that was seen from in vivo bioluminescence imaging at day 21. However, these results could not demonstrate the anti-tumor effect of FLP via inhibition of Cox-2 expression since FLP ointment only effectively inhibited Cox-2 expression at day 14. In addition, *vivo* bioluminescence imaging experiment might replace regularly sacrificed mice in the observation of the tumor changes.

Reducing tumor-associated inflammation and preventing recurrence and metastasis are crucial therapeutic strategies for the management of cancer patients. Chinese medicine practitioners believe that the biggest advantage of TCM is not only the inhibition of tumor growth, but also the prevention of tumor recurrence and metastasis. According to the basic theory of TCM, the concept of holism in TCM is consistent with the tumor microenvironment just as macroscopical and microscopical aspects. Therefore, we hypothesized that the mechanism of TCM in preventing tumor recurrence and metastasis rely on regulation of the tumor microenvironment [31]. The crucial event in the course of lung cancer is its metastatic spread to distant organs. Metastatic cancer cells (‘seeds’) arise from the primary tumors need to create a specific organ microenvironments (‘soil’), which is called organ targeting [48].The tumor microenvironment can be divided into three categories: core of primary tumor microenvironment, invasive tumor microenvironment, and metastatic tumor microenvironment [5]. We hypothesized that TCM therapies might prevent the affinity of “seed and soil” through regulating the tumor metastatic microenvironment and preventing tumor recurrence and metastasis. The current study also demonstrated increased Cox-2 expression in the lung metastatic microenvironment and that the inhibitory effect of FLP ointment on Cox-2 expression was strengthened over time. This suggested that TCM therapies reduce the risk of lung metastasis through effective regulation of the metastatic inflammatory microenvironment.

Previous studies have suggested that celecoxib inhibits Cox-2 expression in a variety of tumors, such as colon cancer, lung cancer, breast cancer, gastric cancer, and prostate cancer, and plays role in regulating tumor occurrence and progression [49–53]. However, the application of Cox-2 inhibitor for the treatment and prevention of cancer remains controversial. Anti-tumor effects of Cox-2 inhibitor alone or in combination with other chemotherapy drugs have been widely investigated, and many trials have shown inspiring results. For example, a Phase II study showed that treatment of celecoxib with cisplatin plus etoposide in extensive-stage small cell lung cancer was safe and feasible [54]. A phase I and pharmacokinetic studies demonstrated an antitumor effect of celecoxib in combination with ocetaxel and irinotecan [55]. The combination of the celecoxib and paclitaxel has been considered as the second-line therapy for non-small cell lung cancer [56]. On the contrary, chemotherapy or targeted drugs in combination with celecoxib did not improve the patient survival or remission rate in two clinical trials [57, 58]. Celecoxib inhibited tumor growth and lung metastasis in Lewis lung carcinoma xenograft C57BL/6 mice, and the mechanism was related to the early inhibition of the Cox-2 expression of the tumor specimens [49]. In our study, FLP ointment in combination with celecoxib exerted significant advantages in inhibiting Cox-2 expression in the lung metastatic microenvironment. The synergistic effect might be partly explained by the celecoxib-mediated stable and continuous inhibition of Cox-2 expression in lung metastatic microenvironment and FLP ointment- mediated inhibition of Cox-2 expression in a time-dependent manner (Fig. 4). FLP ointment inhibited Cox-2 and metastasis- related downstream proteins. FLP ointment was superior to celecoxib in inhibiting MMP-9 and Vimentin protein expression. FLP might regulate these pathways via secondary inhibition of upstream pathways, or by directly inhibiting target protein expression. At the same, FLP ointment in combination with celecoxib synergistically inhibited angiogenesis and EMT or ECM- related protein expression.

To the best of our knowledge, no previous literature has reported the combined use of TCM and celecoxib in the treatment and prevention of cancer. Our findings could provide some basis for future clinical trials. Further studies are warranted to elucidate whether FLP ointment in combination with celecoxib can clinically provide the best efficacy in the prevention of recurrence and metastasis.

## Conclusions

This study demonstrated that FLP ointment is effective in regulating the metastatic inflammatory microenvironment from multi-target via inhibition of Cox-2 expression and regulating lung metastasis-related proteins. Treatment of FLP ointment in combination with celecoxib adjunctly inhibits tumor growth and lung metastasis in a Lewis lung carcinoma xenograft mouse model. TCM combined with anti-inflammatory drugs might be a promising strategy in the prevention of tumor metastasis. Moreover, TCM might be a potential candidate drug of maintenance therapy. Further studies on anti-tumor and anti-metastatic effect of TCM may significantly contribute to the management of lung cancer patients.

## Abbreviations

FLP: Fei-Liu-Ping
Cox: Cyclooxygenase
MMP: metalloproteinases
PDGFR: platelet-derived growth factor receptors
EMT: epithelial-mesenchymal transition
ECM: extracellular matrix
NSAIDs: non steroidal anti-inflammatory drugs
TCM: Traditional Chinese Medicine
VEGF: Vascular endothelial growth factor
ROI: region of interest
PGE2: Prostaglandin E2
mPGES-1: microsomal Prostaglandin E synthase-1
